# Non-Steroidal Anti-Inflammatory Drugs Use Is Associated with Reduced Risk of Inflammation-Associated Cancers: NIH-AARP Study

**DOI:** 10.1371/journal.pone.0114633

**Published:** 2014-12-31

**Authors:** Fatma M. Shebl, Ann W. Hsing, Yikyung Park, Albert R. Hollenbeck, Lisa W. Chu, Tamra E. Meyer, Jill Koshiol

**Affiliations:** 1 Division of Cancer Epidemiology and Genetics, National Cancer Institute, NIH, Department of Human Health and Services, Rockville, Maryland, United States of America; 2 AARP (Retired), Washington, District of Columbia, United States of America; 3 Cancer Prevention Institute of California, Fremont, California, United States of America; 4 Stanford Cancer Institute, Palo Alto, California, United States of America; University of Calgary, Canada

## Abstract

**Background:**

Chronic inflammation has been linked to cancers, and use of non-steroidal anti-inflammatory drugs (NSAIDs) has been associated with reduced risk of several cancers. To further refine the magnitude of NSAID-related associations, in particular for cancers related to inflammation, such as alcohol-, infection-, obesity-, and smoking-related cancers, as well as for less common cancers, we evaluated the use of NSAIDs and cancer risk in a very large cohort. We used propensity scores to account for potential selection bias and hypothesized that NSAID use is associated with decreased cancer incidence.

**Methods:**

We conducted a prospective study among 314,522 participants in the NIH-AARP Diet and Health Study. Individuals who completed the lifestyle questionnaire, which included NSAID use, in 1996–1997 were followed through 2006. Information on cancer incidence was ascertained by linking to cancer registries and vital status databases.

**Findings:**

During 2,715,994 person-years of follow-up (median 10.1 person-years), there were 51,894 incident cancers. Compared with non-users of NSAIDs, individuals who reported use in the 12 months prior to interview had a significantly lower risk of all inflammation-related cancer, alcohol-related, infection-related, obesity-related, and smoking-related cancers [hazard ratio (HR) (95% CI)) 0.90 (0.87–0.93), 0.80 (0.74–0.85), 0.82 (0.78–0.87), 0.88 (0.84–0.92), and 0.88 (0.85–0.92) respectively)].

**Conclusions:**

After accounting for potential selection bias, our data showed an inverse association between NSAID use and alcohol-related, infection-related, obesity-related, and smoking-related cancers and support the hypothesis that inflammation is related to an increased risk of certain cancers.

## Introduction

Inflammation has been linked to several cancers, including cancers of the colon, liver, stomach, and gallbladder. Inflammation is involved in cancer initiation, progression, angiogenesis, invasion, and metastasis and can be prompted by several factors, including infection, smoking, alcohol use, and obesity [1]–[4]. Therefore, it is plausible that NSAIDs could attenuate the risks of cancers for which these causes of inflammation are risk factors.

Non-steroidal anti-inflammatory drug (NSAID) use, especially of aspirin, has been linked to reduced risk of cancers in several, [5]–[8] but not all [5]–[11] observational studies. Data from clinical trials of NSAIDs have shown that NSAID use can lower ovarian and colorectal cancer risk [12]–[15]. However, the role of NSAID use in less common cancers is unclear due to the small numbers of these cancers in previous studies. In addition, cancers that have inflammation-related causes in common have not been jointly evaluated. Evaluating these cancers as a group could help eliminate some of the uncertainty from previous studies and elucidate the role of NSAIDs in inflammation-related cancers.

Our objective is to investigate the role of NSAIDs use in less common cancer and cancers with related inflammatory causes (i.e., obesity, infection, alcohol, and smoking). Therefore, we examined the association between incident cancer and the self-reported aspirin and non-aspirin NSAIDs in a large (>300,000 subjects), well-designed study: the National Institute of Health-AARP (NIH-AARP) Diet and Health Study cohort [16]. Given the large size of the NIH-AARP cohort, we were well-powered to investigate the role of NSAID in several less common and inflammation-related cancers.

## Subjects and Methods

### Study population

The NIH-AARP cohort was established in 1995–1996 as described elsewhere [16]. In brief, questionnaires were mailed to 50–71-year-old AARP members in two metropolitan areas (Detroit, Michigan, and Atlanta, Georgia) and six U.S. states (Pennsylvania, New Jersey, North Carolina, Louisiana, Florida, and California); 18% returned the baseline questionnaire. A subsequent questionnaire requesting additional risk factor data, including NSAID use, was mailed six months later to participants without self-reported colon, breast or prostate cancer in the baseline questionnaire.

### Ethical approval

Ethical approval of the NIH-AARP Diet and Health Study was granted by the National Cancer Institute's Special Studies Institutional Review Board. Participants mailed the written consent and the study materials to the study team.

For the current analysis, we excluded proxies (n = 10,383), individuals with cancer ascertained in the death report only (n = 1,786), individuals with missing NSAID use data (n = 3,634), and those with no follow-up (n = 40). Our analytic NIH-AARP cohort included 314,522 persons (132,462 men and 182,060 women) with data from the baseline and risk factor questionnaires.

### Cohort follow-up and cancer ascertainment

#### Identification of cancer cases

The study linked cohort members to state cancer registry databases in the original eight states and three additional states (AZ, NV, and TX) where participants tended to move during follow-up. The case ascertainment method identified approximately 90% of all cancer cases in our cohort [17]. In order to update participants' vital status, annual linkage of the Social Security Administration Death Master and the cohort were attained. Further confirmation of participants' vital status was verified by other methods including; matching the Social Security Administration Death Master File with the National Death Index Plus, mailings, and questionnaire responses.

### NSAID use assessment

Participants were asked about their use of aspirin (e.g. Anacin, Bayer, Bufferin, Ecotrin, Excedrin, or generic aspirin) and non-aspirin (e.g. Advil, Aleve, Anaprox, Clinoril, Feldene, Fenoprofen, generic ibuprofen, Indocin, Indomethacin, Ketoprofen, Motrin, Naprosyn, Nalfon, Nambumetone, Nuprin, Orudis, Piroxicam, Relafen, or Sulindac) pain relievers during the past 12 months. When asked about non-aspirin NSAIDs, participants were directed not to include acetaminophen, Tylenol, or any other non-NSAID pain relievers. For individuals reporting NSAID use in the year prior to the risk-factor questionnaire, we defined the frequency of aspirin and non-aspirin NSAID use as follows: monthly (≤3 times/month), weekly (≥1 times/week) and daily (≥1 times/day). In the analysis, use of NSAIDs, aspirin, or non-aspirin was defined as yes/no while frequency of use was defined as never, monthly, weekly or daily. In addition, we created a 4-category variable defining type of NSAID use (no NSAID, aspirin-only, non-aspirin-only, both aspirin and non-aspirin).

### Statistical analysis

All analyses were conducted using SAS/STAT software, Version 9.2 (SAS Institute Inc., Cary, NC, USA). We assessed the association between incident inflammation-related cancers and the use of any NSAIDs, aspirin, and non-aspirin NSAIDs. We used non-users of any NSAIDs as the reference group in all analyses.

We assessed overall inflammation-related cancer risk, and risk for cancers with specific inflammation-related causes, explicitly: obesity-related cancers (esophageal, gallbladder, colorectal, pancreatic, post-menopausal breast, endometrial, kidney, and thyroid); infection-related cancers (head and neck, stomach, liver, colorectal, lymphoma, anal, and female genital) [18], alcohol-related cancers (head and neck, esophageal, colorectal, liver and breast) [19], [20], and smoking-related cancers (lung, head and neck, esophageal, pancreatic, and urinary bladder) [21]. To avoid reverse causality, we excluded cancer cases that were reported during the first year of follow-up and conducted sensitivity analysis excluding cancers observed in the first five years. Although we lacked information on the indication for NSAID use, we assessed the potential for indication bias by examining NSAID usage among non-diabetics and individuals without cardiovascular disease history.

Since individuals were not randomized to NSAID use, NSAID users might be systematically different from non-users, leading to selection bias. To minimize selection bias and mimic experimental design, we used the propensity score stratification method [22]. The propensity score is the likelihood of NSAID use, which was calculated using logistic regression such that NSAID use was regressed on baseline characteristics (Table 1). Accordingly, data were divided into five approximately equal strata using the calculated propensity scores. Because the propensity score should act as a balancing score, we used the standardized difference method [23] to examine whether propensity score stratification balanced the distribution of variables used in calculating propensity score among NSAID users and non-users in the five strata. The standardized difference was calculated as the difference in means/proportion of the variables in NSAID and non-NSAID users divided by the standard deviation of the variable. To estimate the effect of NSAID use on cancer risk, we used propensity score stratified proportional hazards Cox regression models for calculating hazard ratios (HRs) and 95% confidence intervals (CIs). Such approach led to weighted HRs, and allowed the baseline hazards to differ among propensity score strata. Since, sex, race, age, education, marital status, family history of cancer, diabetes, alcohol use, smoking, self-reported history of cardiovascular disease, and body mass index (BMI) were used in propensity score calculation, these variables were not included in the Cox models except if a variable was not balanced after propensity score stratification. By using propensity score stratification, we minimize potential bias due to different distribution of these variables among NSAID and non-NSAID users. Additional covariates that were specific to individual types of cancer were added to the Cox models as appropriate for that cancer. For example, prostate cancer was adjusted for prostate-specific antigen (PSA) testing, and breast cancer was adjusted for parity, age at first birth, age at menopause, hormonal replacement therapy (HRT) use, HRT duration, hysterectomies, and oophorectomies. To adjust for multiple comparisons we used false discovery rate (FDR) method. FDR adjusted p values were calculated allowing not more than 0.2 true null hypothesis [24]. Trend was assessed by including the frequency of NSAID use as a continuous variable that ranges from 0 to 3, where 0 is never used, 1 is monthly use, 2 is weekly use and 3 is daily use.

**Table 1:** Baseline characteristics of participants in NIH-AARP Diet and Health Study.

		No cancer	Cancer	No NSAIDs use	NSAIDs use
N		262,628	51,894	272288	42234
Age (mean, SD)		62.6 (5.4)	64.1 (4.9)	63.7 (5.1)	62.7 (5.3)
Person years (mean, SD)		9.4 (2.1)	5.0 (2.9)	8.5 (2.8)	8.7 (2.7)
Sex (n, %)	Female	147,564 (56.2)	33,496 (64.5)	22,267 (52.7)	159,784 (58.7)
	Male	115,064 (43.8)	17,398 (33.5)	19,958 (47.3)	112,504 (41.3)
Race/ethnicity (n, %)	White, non-Hispanic	242,680 (92.4)	48,532 (93.5)	37642 (90.3)	253570 (94.2)
	Black, non-Hispanic	8,684 (3.3)	1,628 (3.1)	2,248 (5.4)	8,064 (3.00)
	Others	8,284 (3.2)	1,150 (2.2)	1,785 (4.3)	7,649 (2.8)
Marital status (n, %)	Married	176,559 (67.2)	36,809 (70.9)	26,466 (63.2)	186,902 (69.1)
Education (n, %)	High school or less	61,500 (23.4)	12,181 (23.5)	12,052 (29.4)	61,629 (23.2)
	Post high school training	259,94(9.9)	4,950 (9.5)	4,283 (10.4)	26,661 (10.0)
	Some college	61,365 (23.4)	12,000 (23.1)	9,477 (23.1)	63,888 (24.1)
	College graduate	107,279 (40.8)	21,377 (41.2)	15,199 (37.1)	113,457 (42.7)
Family history of cancer (n, %)	Yes	129,722 (49.4)	27,057 (52.1)	20,765 (51.5)	136,014 (52.3)
History of disease (n, %)	Diabetes	22,245 (8.5)	4,555 (8.8)	4,031 (9.5)	22,769 (8.4)
	Heart disease	35,469 (13.5)	8,083 (15.6)	5,269 (12.5)	38,283 (14.1)
Body mass index (n, %)	<25	95,987 (36.5)	17,979 (34.6)	16,730 (40.7)	97,236 (37.4)
	25-<30	106,892 (40.7)	22,236 (42.8)	16,059 (39.1)	113,069 (42.3)
	30-<35	38,771 (14.8)	7,739 (14.9)	5,725 (13.9)	40,785 (15.3)
	35+	15,627 (6.0)	2,907 (5.6)	2,570 (6.3)	15,964 (6.0)
Smoking (n, %)	Never	96,873 (36.9)	15,478 (29.8)	16,336 (40.0)	96,015 (36.5)
	Former	74,136 (28.2)	14,143 (27.3)	19,634 (48.1)	137,306 (52.2)
	Current	55,462 (21.1)	13,199 (25.4)	4,850 (11.9)	29,902 (11.3)
Alcohol (n, %)	None	19,495 (7.4)	3,448 (6.6)	4,742 (11.2)	18,201 (6.7)
	<5 g/day	147,447 (56.1)	27,196 (52.4)	24,882 (58.9)	149,761 (55.0)
	5-<15 g/day	40,967 (15.6)	8,277 (15.9)	5,124 (12.1)	44,120 (16.2)
	15-<30 g/day	28,374 (10.8)	6,228 (12.0)	3,652 (8.7)	30,950 (11.4)
	30+ g/day	26,345 (10.0)	6,745 (13.0)	3,834 (9.1)	29,256 (10.7)

## Results

### Population characteristics

During 2,715,994 person-years of follow-up (median 10.1, inter-quartile range 8.7–10.1 years), 51,894 individuals answered the NSAID use questions in the lifestyle questionnaire developed cancer, and 262,628 did not. The baseline mean age of study participants was 62.9 (SD 5.3) years; 57.9% were male, and 93.7% were white. Diabetes, heart disease, stroke, hypertension, and ever smoking were reported by 8.5%, 13.9%, 1.9%, 43.4% and, 63.0%, respectively (Table 1). Approximately 86.5% of the subjects reported using NSAIDs; 30% used only aspirin, 13.4% used only non-aspirin NSAIDs, and 43.0% used both aspirin and non-aspirin NSAIDs.

### Use of any NSAID, any aspirin, and any non-aspirin NSAID

Risk of all inflammation-related cancers was reduced in association with the use of any NSAID (HR 0.90, 95% CI 0.87–0.93), aspirin (HR 0.94, 95% CI 0.92–0.97), and non-aspirin (HR 0.93, 95% CI 0.91–0.95) (Table 2). Risks of alcohol-related, infection-related, obesity-related, and smoking-related cancers were also reduced with NSAID use. Aspirin (regardless of non-aspirin NSAID use) was associated with reduced risk of infection-related and obesity-related cancers. Non-aspirin NSAIDs (regardless of aspirin use) were significantly associated with a lower risk of alcohol-related, infection-related, obesity-related, and smoking-related cancers (Table 2).

**Table 2:** Hazard ratios and 95% confidence intervals for cancer risk in relation to NSAID, aspirin and non-aspirin use in the past 12 months from the NIH-AARP Diet and Health Study*.

	Use of NSAIDs	Use of aspirin	Use of non-aspirin
All inflammation related cancers	0.90 (0.87–0.93)	0.94 (0.92–0.97)	0.93 (0.91–0.95)
Alcohol-related cancer&	0.80 (0.74–0.85)	0.95 (0.90–1.01)	0.78 (0.74–0.82)
Infection-related cancer$	0.82 (0.78–0.87)	0.92 (0.88–0.96)	0.84 (0.81–0.88)
Obesity-related cancer#	0.88 (0.84–0.92)	0.89 (0.83–0.97)	0.95 (0.92–0.98)
Smoking-related cancer^∧^	0.88 (0.85–0.92)	0.98 (0.91–1.00)	0.88 (0.86–0.90)

* Three models each for one of the variables including use of NSAID, use of aspirin, or use of non-aspirin.

& Head and neck, esophagus, colorectal, liver and breast cancer for women.

$ Head and neck, stomach, liver, colorectal, lymphoma, and female genital cancers.

# Esophagus, gallbladder, colorectum, pancreas, breast (after menopause), endometrium, kidney, and thyroid.

^∧^ Lung, head and neck, esophagus, pancreas, and urinary bladder cancers.

For individual cancers, we also observed that overall NSAIDs were associated with significantly reduced risk of esophageal (HR 0.74, 95% CI 0.58–0.95), stomach (HR 0.73, 95% CI 0.58–0.93), liver (HR 0.59, 95% CI 0.44–0.78), colorectal (HR 0.79, 95% CI 0.73–0.86), prostate (HR 0.94, 95% CI 0.89–0.99), endometrial (HR 0.77, 95% CI 0.65–0.92), and lung (HR 0.89, 95% CI 0.83–0.96) cancers (data not in tables). Aspirin was associated with reduced risk of cancers of the liver (HR 0.62, 95% CI 0.49–0.79), and endometrium (HR 0.86, 95% CI 0.75–0.99) but the excess risk of urinary bladder cancers (HR 1.16, 95% CI 1.05–1.27). Non-aspirin was associated with reduced risk of cancers of the esophagus (HR 0.74, 95% CI 0.62–0.89), stomach (HR 0.70, 95% CI 0.58–0.84), pancreas (HR 0.87, 95% CI 0.77–0.98), colorectum (HR 0.75, 95% CI 0.71–0.80), head and neck (HR 0.87, 95% CI 0.77–0.97), lung (HR 0.94, 95% CI 0.89–0.99), urinary bladder (HR 0.88, 95% CI 0.81–0.95), and myeloid monocytic leukemia (HR 0.77, 95% CI 0.63–0.94), prostate (HR 0.94, 95% CI 0.91–0.97), and endometrium (HR 0.87, 95% CI 0.76–1.00) (data not in tables).

### Independent and combined effects of aspirin and non-aspirin NSAID use

**Figure 1:**
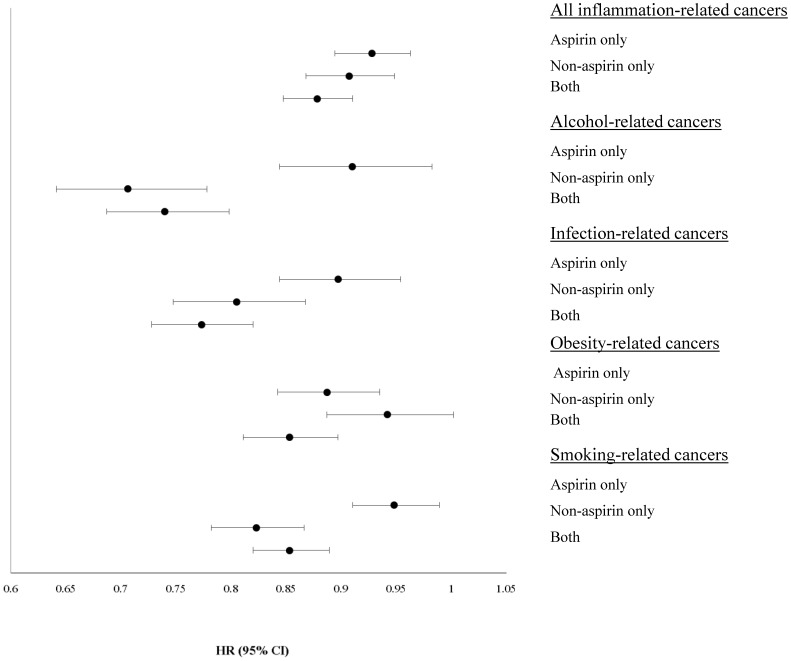
Hazard ratios and 95% confidence interval for cancer risk in relation to exclusive use of Aspirin or non-aspirin NSAID, or use of both NSAISDs in the past 12 months from the NIH-AARP Diet and Health Study*. *NSAID use is a four-level variable, of NSAID non-use, use of aspirin alone, use of non-aspirin NSAIDs alone, or use of both aspirin and non-aspirin NSAIDs. NSAID non-use is the reference. # Esophagus, gallbladder, colorectum, pancreas, breast (after menopause), endometrium, kidney, and thyroid. $ Head and neck, stomach, liver, colorectal, lymphoma, and female genital cancers. & Head and neck, esophagus, colorectal, liver and breast cancer for women. ^∧^Lung, head and neck, esophagus, pancreas, and urinary bladder cancers. HR: hazard ratio. 95% CI: 95% confidence interval.

In addition to evaluating the use of any NSAID, aspirin, and non-aspirin, we examined the use of aspirin alone, use of non-aspirin NSAIDs alone, and use of both aspirin and non-aspirin NSAIDs (Fig. 1). Compared with non-users, aspirin-only, non-aspirin-only, and both aspirin and non-aspirin NSAID users had lower risks of all inflammation-related, alcohol-related, infection-related, obesity-related and smoking-related cancers.

Models for specific cancer sites showed that aspirin-only users had reduced risks of liver (HR 0.52, 95% CI 0.37–0.73), lung (HR 0.91, 95% CI 0.84–0.99), and skin (HR 0.88, 95% CI 0.78–0.98) cancers, but excess risk of urinary bladder (HR 1.18, 95% CI 1.04–1.35) cancers (data not in tables). In addition, non-aspirin NSAID-only users had reduced risks of esophageal (HR 0.70, 95% CI 0.50–0.99), stomach (HR 0.66, 95% CI 0.47–0.93), colorectal (HR 0.68, 95% CI 0.60–0.76), endometrial (HR 0.76, 95% CI 0.61–0.95), lung (HR 0.81, 95% CI 0.73–0.90), and any skin (HR 0.79, 95% CI 0.69–0.91) cancers, including melanoma (HR 0.80, 95% CI 0.69–0.93). Users of both aspirin and non-aspirin had reduced risk of esophageal (HR 0.66, 95% CI 0.50–0.86), stomach (HR 0.65, 95% CI 0.50–0.84), liver (HR 0.58, 95% CI 0.42–0.79), colorectal (HR 0.73, 95% CI 0.66–0.79), lung (HR 0.90, 95% CI 0.83–0.98), myeloid monocytic leukemia (HR 0.70, 95% CI 0.51–0.94), and endometrial cancers (HR 0.73, 95% CI 0.60–0.88) (data not in tables).

### Frequency of NSAID use

As shown in Table 3, inverse dose response trends were observed for infection-related and obesity-related cancers with frequency of aspirin use compared to non-use (p_trend_<0.001). Interestingly, smoking-related cancers exhibited a positive dose response relationship with aspirin use compared to non-use (p_trend_<0.001). For the frequency of non-aspirin NSAID use, an inverse trend was observed for all inflammation-related (p_trend_<0.001), alcohol-related (p_trend_<0.001), infection-related (p_trend_<0.0001), and smoking-related (p_trend_<0.001) cancers. None of the examined cancers showed a significant inverse dose response association with aspirin use compared to non-use (data not in tables). However, cancers of the esophagus, stomach, pancreas, colorectum, endometrium, head and neck, urinary bladder, leukemia, and NHL all had significant inverse dose response trend with non-aspirin NSAD use compared to non-use.

**Table 3:** Hazard ratios and 95% confidence intervals for cancer risk in relation to frequency of aspirin and non-aspirin use and cancer in the NIH-AARP Diet and Health Study.

	Monthly	Weekly	Daily	P for trend*
***Aspirin use***				
All inflammation related cancers	0.92 (0.89–0.95)	0.92 (0.89–0.96)	0.99 (0.96–1.02)	0.64
Alcohol-related cancer&	0.95 (0.89–1.01)	0.89 (0.82–0.96)	1.01 (0.94–1.08)	0.87
Infection-related cancer$	0.92 (0.87–0.97)	0.89 (0.83–0.94)	0.93 (0.88–0.98)	<0.01
Obesity-related cancer#	0.92 (0.88–0.96)	0.89 (0.84–0.93)	0.86 (0.82–0.90)	<0.001
Smoking-related cancer^∧^	0.93 (0.90–0.96)	0.94 (0.90–0.98)	1.08 (1.04–1.12)	<0.001
***Non-aspirin use***				
All inflammation related cancers	0.92 (0.90–0.95)	0.93 (0.89–0.96)	0.96 (0.92–1.00)	<0.001
Alcohol-related cancer&	0.83 (0.78–0.88)	0.74 (0.68–0.81)	0.70 (0.64–0.77)	<0.001
Infection-related cancer$	0.86 (0.82–0.90)	0.82 (0.77–0.87)	0.81 (0.75–0.87)	<0.001
Obesity-related cancer#	0.94 (0.90–0.99)	0.97 (0.92–1.02)	0.96 (0.90–1.01)	0.05
Smoking-related cancer^∧^	0.89 (0.86–0.92)	0.85 (0.81–0.88)	0.88 (0.84–0.93)	<0.001

*FDR adjusted p values did not appreciably differ from the non-FDR adjusted p values.

& Head and neck, esophagus, colorectal, liver and breast cancer for women.

$ Head and neck, stomach, liver, colorectal, lymphoma, and female genital cancers.

# Esophagus, gallbladder, colorectum, pancreas, breast (after menopause), endometrium, kidney, and thyroid.

^∧^ Lung, head and neck, esophagus, pancreas, and urinary bladder cancers.

Prior to examining the association between NSAIDs use and cancer adjusting for confounders, we examined whether gender modified the risk. Because no effect modification was detected except for the infection-related cancer and ever use of aspirin, and obesity-related cancer with ever use of ibuprofen, we did not present all analyses stratified by gender. Of note, the association between infection-related cancer and ever use of aspirin was significant among women (HR 0.87, 95% CI 0.82–0.93), but not among men (HR 0.96, 95% CI 0.90–1.02). On the other hand, while obesity-related cancer was significantly reduced among women and men who reported ever use of ibuprofen, however the effect was more profound among women (HR 0.85, 95% CI 0.80–0.90), compared to men (HR 0.91, 95% CI 0.88–0.95).

### Sensitivity analyses

The results among non-diabetics and individuals with no cardiovascular disease history were similar to those for the overall analysis. Among non-diabetics, use of any NSAID was associated with a reduced risk of inflammation-related (HR 0.89, 95% CI 0.86–0.93), alcohol-related (HR 0.79, 95% CI 0.73–0.85), infection-related (HR 0.81, 95% CI 0.77–0.86), obesity-related (HR 0.88, 95% CI 0.84–0.92), and smoking-related cancers (HR 0.87, 95% CI 0.84–0.92). Among individuals without history of cardiovascular diseases, use of any NSAID was associated with a reduced risk of inflammation-related (HR 0.92, 95% CI 0.88–0.96), alcohol-related (HR 0.81, 95% CI 0.73–0.89), infection-related (HR 0.85, 95% CI 0.79–0.82), obesity-related (HR 0.93, 95% CI 0.87–0.99) and smoking-related cancers (HR 0.88, 95% CI 0.84–0.93).

Since some anti-cholesterol medication might affect cancer risk, we conducted a sensitivity analysis restricted to individuals who had information on the frequency of anti-cholesterol medication within last year. Our results revealed that the overall risk of cancer (HR 0.92, 95% CI 0.89–0.96), inflammation-related (HR 0.90, 95% CI 0.86–0.94), alcohol-related (HR 0.77, 95% CI 0.70–0.84), infection-related (HR 0.80, 95% CI 0.75–0.86), obesity-related (HR 0.88, 95% CI 0.83–0.93) and smoking-related cancers (HR 0.87, 95% CI 0.82–0.91) remained significantly reduced after adjusting for anti-cholesterol medication. Interestingly, the frequency of use of anti-cholesterol medication was significantly associated with excess overall cancer risk (HR 1.028, 95% CI 1.024–1.033), inflammation-related (HR 1.027, 95% CI 1.020–1.033), alcohol-related (HR 1.032, 95% CI 1.018–1.046), and smoking-related cancers (HR 1.055, 95% CI 1.047–1.063).

We further conducted a sensitivity analysis to assess whether excluding the first five years of follow up would change our findings. The risk of inflammation-related cancer in association with any NSAID (HR 0.90, 95% CI 0.86–0.95), aspirin (HR 0.94, 95% CI 0.91–0.98), and non-aspirin use (HR 0.93, 95% CI 0.90–0.96) did not appreciably change.

## Discussion

In this large prospective study, we conducted extensive analyses of NSAID use and cancer that expanded on previous studies by 1) evaluating groups of cancers with common inflammation-related causes and 2) accounting for self-selection bias through propensity scores. By combining cancers with similar causes, we could better evaluate non-aspirin NSAID use, which has a lower prevalence than aspirin use. The use of propensity scores provides increased confidence in these results given concerns that previously observed inverse associations may be largely due to selection bias [25].

We showed that NSAID use was associated with a reduced risk of cancers that usually develop in the presence of inflammatory conditions such as obesity, smoking, infection, and excessive alcohol consumption. Aspirin use was associated with reduced risk of infection-related, obesity-related, liver, and endometrial cancers. Non-aspirin NSAID use was associated with lower risk of inflammation-related, alcohol-related, infection-related, obesity-related, smoking-related, head and neck, esophageal, stomach, pancreas, colorectal, lung, urinary bladder, endometrial and prostate cancers, and myeloid monocytic leukemia. More specifically, aspirin-only users had reduced risks of liver, lung, skin, and endometrial cancers but the excess risk of urinary bladder cancers. Non-aspirin NSAID-only users had a reduced risk of esophageal, stomach, colorectal, prostate, skin, melanoma, and lung. Both aspirin and non-aspirin NSAID use were associated with lower risk of all inflammation-related, alcohol-related, infection-related, obesity-related, and smoking-related cancers, and individual cancers including esophageal, stomach, liver, colorectal, endometrial, and lung cancers.

Overall, our results support the role of NSAIDs in reducing inflammatory conditions and thus diminishing risk of inflammation-related cancers [3]. This role is plausible, given the known mechanisms of action of NSAIDs. The main mechanism involves inhibition of COX enzymes leading to suppression of prostaglandin synthesis, which subsequently modulates cellular proliferation and apoptosis, hindering tumor growth [26]–[28]. Alternative COX-independent anti-neoplastic mechanisms include MAGI1, NF-κB, TGF-β, RAS, β-catenin, and cyclin-D1 pathways [29]–[32].

Our findings are further supported by studies of individual cancers. The observed reduced risk of alcohol-related cancers in association with NSAID use is supported by studies of individual alcohol-related cancers, such as head and neck, esophagus, colorectal, and breast [33]–[37]. Similarly, NSAID use has been associated with reduced risk of obesity-related cancers, including esophageal, gallbladder, colorectal, pancreatic, post-menopause breast, endometrial, kidney, and thyroid cancers [35], [38], [39]. NSAID use is also associated with decreased risk of individual infection-related cancers, specifically head and neck, stomach, liver, colorectal, lymphoma, and female genital cancers [33], [35], [40]. Finally, studies of NSAIDs and; lung, head and neck, esophagus, pancreas, and urinary bladder cancers [5], [41]–[45] support our finding of reduced risk of smoking-related cancer.

Our findings of lower risks at specific cancer sites are also consistent with results from previous studies [5]–[8], [46], [47] and several meta-analyses [6], [35], [41], [43], [48]–[52] demonstrating lower risks of including esophageal, gastric, colorectal, and lung cancers associated with aspirin use. Similarly, the observed reduced risk of stomach, colorectal cancers in association with non-aspirin NSAIDs are supported by epidemiologic and in vitro studies [53]–[55]. For gastric cancer risk a study from Taiwan found that NSAID use was protective against gastric cancer risk even among individuals with *Helicobacter pylori* infection [56]. A study of the effect of aspirin in the presence of *Helicobacter pylori* revealed that aspirin increases *Helicobacter pylori*-induced apoptosis and diminish *Helicobacter pylori*-induced hyperplasia [57].

Reduced esophageal cancer risk has been associated with both aspirin and non-aspirin NSAID use [34], [43], [58], [59]. Similarly, the lower risk of colorectal cancer among NSAID users has been observed in previous studies [46], [53], [60]–[62], even among individuals with previously treated colorectal cancer [15].

The association of NSAID use with reduced liver cancer in human is a fairly novel finding, as described in a separate AARP publication [25]. Liver cancer risk factors include chronic hepatitis virus infection, alcohol, and obesity, all of which involve chronic inflammation mediated by several molecular pathways [63]. Recently, statin use was reported to diminish HCC risk, a further support of the role of anti-inflammatory agents in reducing HCC risk [64]. In addition, in a recent study of a nude mouse xenograft model, aspirin repressed growth of hepatocellular carcinoma cells and induced apoptosis in vitro [65]. Also, aspirin was found to increase reactive oxygen species production and induce cell cycle arrest and apoptosis in HepG2 cells [25], again supporting the hypothesis that NSAID use may lower the risk of liver cancer by reducing inflammation.

The association between lung cancer and NSAID use has been inconsistent in previous studies. Some studies supported the diminished risk, while other studies failed to detect reduced risk, or found an effect among men, but not women, or an effect in specific histologic subtypes [19], [23], [64], [66]–[68]. However, two large studies found an inverse association between NSAID use and lung cancer [23], [67], as did a recent pooled analysis from the International Lung Cancer Consortium (ILCCO), which reported a 26% risk reduction in men [6]. These studies support our results. Conflicting results from other studies might be attributed to lack of power due to small sample size, residual confounding due to incomplete smoking information, or inadequate NSAID dose and duration information.

In concordance with our findings, a lower risk of myeloid leukemia in association with non-aspirin use has been reported [69], [70]. This association is further supported by in vitro studies of the effect of NSAIDs on inducing apoptosis in acute myeloid leukemia cells [71].

Studies of NSAID use and melanoma have conflicting results. Our finding of reduced risk with non-aspirin-only use, but not with aspirin is interesting. A few studies reported reduced risk of melanoma [72]–[74] including a recent large population-based study from Northern Denmark [75]. On the other hand, some studies reported excess, although non-significant, risk of melanoma with non-aspirin use [76], [77]. A large Dutch population-based study reported a non-significant excess risk with non-aspirin NSAIDs but lower risk with continuous use of low-dose aspirin among women only [74]. Although we found a lower risk of endometrial cancer with non-aspirin NSAID use and use of aspirin and non-aspirin NSAIDs together, we did not detect any dose response effect. Previous reports have conflicting results. While some studies reported lower endometrial cancer risk among obese women using NSAIDs [78], [79] other studies failed to detect any association [80], [81]. Our inability to find significant associations between any NSAID use and some cancers might be due to the lack of information on duration of long-term NSAID use [53], [82], [83]. For instance, a cohort study of 70,144 men demonstrated that the current aspirin or non-aspirin use was not associated with lower prostate cancer risk. However, consistent, long-duration use of non-aspirin NSAIDs (≥30 pills/month for ≥5 years) was found to be related to a reduced risk of prostate cancer (relative risk 0.82, 95% CI (0.71 to 0.94). In addition, three large UK trials found that a daily use of aspirin only led to observable reductions in deaths due to several cancers after five years of follow-up, and benefit seemed to increase with duration of treatment [84]. These data suggest that the duration of use may be important, and information on duration and consistency of use was limited in this study. That said, inflammation may contribute more to some cancers/categories of inflammation-related cancers and less to other cancers/categories of inflammation-related cancers, which could explain why we see an association for some cancers/category of inflammation-related cancers and not others.

Evaluation of non-aspirin NSAID use alone was further complicated by the general lower frequency of non-aspirin NSAID use in this population: merely 13.4% used non-aspirin-only. Because aspirin is the most commonly used drug for cardiovascular disease prevention, this study might lack the power to detect diminished risks associated with non-aspirin NSAID use. However, we did improve the power by combining cancers with like inflammation-related causes.

Our study had several strengths. The NIH-AARP Diet and Health Study is a large cohort with detailed information on NSAID use, which allowed us to evaluate less common cancers. The detailed collection of epidemiologic information enabled us to examine the effect of multiple confounders, such as diabetes, smoking, obesity, and cardiovascular diseases. However, certain limitations have to be noted. We did not have information on the indication for NSAID use. However, our results were similar when restricted to individuals without a history of cardiovascular disease and non-diabetics, suggesting that use of NSAIDs for cardiovascular disease does not modify the results. Finally, cumulative exposure or dosage could not be evaluated.

In summary, our results indicate that NSAID use might reduce the risk of several cancers. The null results for some cancers might indicate that NSAIDs need to be used for a prolonged duration to exert a measurable effect. Taken together, these results warrant further studies on the dosage and duration of NSAID use for chemoprevention of inflammation-related cancer. Such studies will pave the way to a well-designed chemoprevention clinical trial to establish the lowest safest dose and duration required for chemoprevention of different cancer subsites.
